# Olfactory cilia, regulation and control of olfaction

**DOI:** 10.14814/phy2.70057

**Published:** 2024-10-02

**Authors:** Hiroko Takeuchi

**Affiliations:** ^1^ Graduated School of Frontier Biosciences Osaka University Suita Osaka Japan

**Keywords:** Cl_(Ca)_ channels, cyclic nucleotide‐gated channels, olfactory adaptation, olfactory cilia, olfactory masking, signal amplification

## Abstract

The sense of smell is still considered a fuzzy sensation. Softly wafting aromas can stimulate the appetite and trigger memories; however, there are many unexplored aspects of its underlying mechanisms, and not all of these have been elucidated. Although the final sense of smell takes place in the brain, it is greatly affected during the preliminary stage, when odorants are converted into electrical signals. After signal conversion through ion channels in olfactory cilia, action potentials are generated through other types of ion channels located in the cell body. Spike trains through axons transmit this information as digital signals to the brain, however, before odorants are converted into digital electric signals, such as an action potential, modification of the transduction signal has already occurred. This review focuses on the early stages of olfactory signaling. Modification of signal transduction mechanisms and their effect on the human sense of smell through three characteristics (signal amplification, olfactory adaptation, and olfactory masking) produced by olfactory cilia, which is the site of signal transduction are being addressed in this review.

## Graphical abstract

Signal modification in cilia and how it affects olfaction.

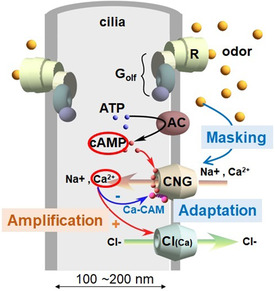

## 1 INTRODUCTION: WHAT ARE THE OLFACTORY CILIA?

Cilia are known as thin pipes with a diameter of 100–200 nm. When most people think of cilia, they imagine “tracheal epithelial cilia” or “primary cilia.” Tracheal epithelial cilia are motor cilia present in large numbers in one cell. They remove foreign substances from the body through their movement (Delmotte & Sanderson, 2006; Fujisawa et al., 2023). In contrast, primary cilia are immobile and are present at one per cell. Their role has gradually become clearer in recent years. They are associated with cell division and cell cycle, and they may function as a signal receptor (Pala et al., 2017). The olfactory cilia are classified as sensory cilia. For example, in sensory cilia, as in photoreceptor cells, there is a region known as the connecting cilium, which connects the outer and inner segments and is associated with retinitis pigmentosa (Mercey et al., 2022). In hair cells involved in hearing, one motor cilium (kinocilium) and multiple immobile cilia (stereocilia) are present in a single cell and function in the initial stage to convert frequencies into electrical signals (Falk et al., 2015; Müller, 2008). Olfactory receptor cells (ORCs) have multiple cilia extending from the tip of the dendrite of one cell into the extracellular mucus layer (Menco & Farbman, 1985; Menco & Morrison, 2003) to convert chemical information carried by odor molecules into electrical signals (Kleene, 2008; Schild & Restrepo, 1998). The number of olfactory cilia ranges from 10 to 20 (Lidow & Menco, 1984; Menco, 1980), and the length is variable depending on the animal species (i.e., 10–50 μm).

During evolution, sensory cells have continued to acquire forms and functions appropriate to their host organism. The same appears to have occurred for the ORCs. Evolutionary biology and sensory physiology studies have shown that olfaction is an old sense, and that during evolution olfaction has been used as the primary sense for recognizing the surrounding environment. Indeed, the major factors involving signal transduction cascades in photoreceptor cells and ORCs are remarkably similar (Bradley et al., 2005; Genovese et al., 2021). Regarding the signal transduction mechanisms in olfactory cilia, the variety and number of olfactory receptor proteins (ORs) differ among animal species; however, the signaling cascades are common from lower vertebrates to mammals (Firestein et al., 1990; Firestein & Werblin, 1987; Grosmaitre et al., 2006; Kurahashi, 1989; Reisert & Matthews, 2001). In this review, signal transduction in olfactory cilia is discussed. An outline of olfactory cilia has been presented previously (Kleene, 2008; Matthews & Reisert, 2003; McClintock et al., 2020; Pifferi et al., 2009). In the present article, the intraciliary molecular movements are also discussed in detail.

## 2 THE SIGNAL TRANSDUCTION SYSTEM IN OLFACTORY CILIA

**FIGURE 1:**
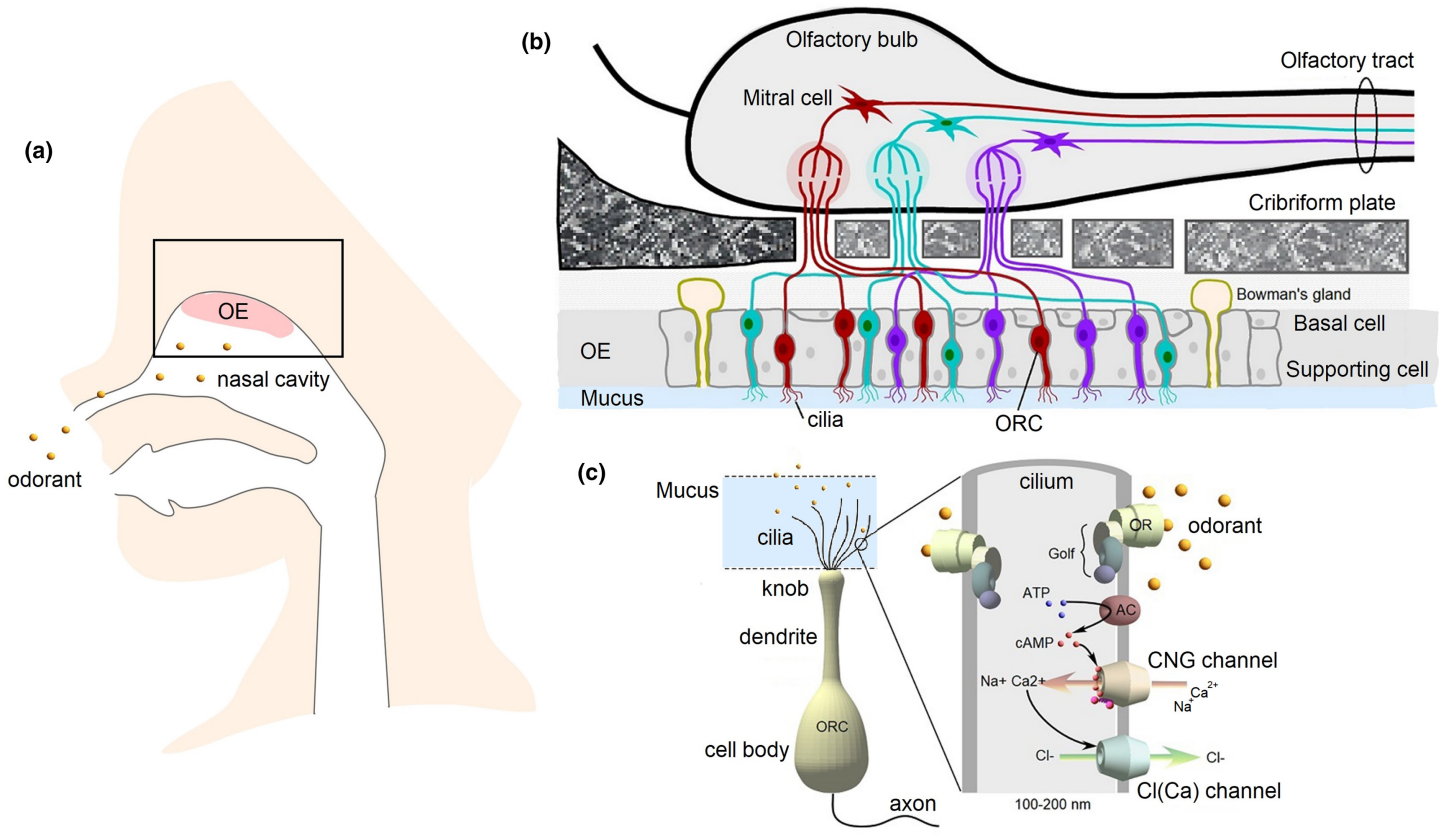
Olfactory perception. (a) Nose and olfactory epithelium (OE) in a sagittal plane of human head. (b) Signal transmission from OE to olfactory bulb (OB). (c) Single olfactory receptor cell (ORC) and a single cilium. Transduction cascade in the cilia. OR, olfactory receptor protein. AC, adenylyl cyclase.

Olfaction occurs through a cascade as shown in Figure 1a–c. External odorants are inhaled into the nasal cavity, where they dissolve into the mucus layer that covers the olfactory epithelial (OE) tissue. The odorants are then transported to the cilia through the mucus and possibly by odorant‐binding proteins (Pelosi et al., 1982; Steinbrecht, 1998), where they bind to ORs expressed on the ciliary membrane. Buck and Axel (1991) discovered the OR genes, which encode proteins containing a putative seven‐transmembrane domain structure. OR genes were found to be highly diverse. Humans have 388 putative functional genes (Niimura & Nei, 2003), and only one of the 388 ORs is expressed in a single ORC (Mombaerts, 2004). In mice, there are approximately 1037 (Niimura & Nei, 2005); however, this large number does not necessarily indicate that the animals are more sensitive to odors. Because there are approximately 6 million ORCs in the human nose (Moran et al., 1982), and each olfactory cell has approximately 10–20 cilia, one can imagine the vast number of ORs expressed on the ciliary membrane, and the number of odorants is thought to be greater than approximately 400,000 (Le Magnen, 1961). Based on a simple statistical calculation, this would allow for 2^388^ patterns, much larger than the hundreds of thousands of odorant types. It is a loose keyhole–key relationship (Buck & Axel, 1991); however, the degree of binding varies greatly depending on the type of key. Because there are hundreds of thousands of odorant types for hundreds of ORs expressed in ORCs, the response pattern is considered “many‐to‐many.”

When an odorant binds to an OR, a cascade is triggered. The ORs are G protein‐coupled receptors (seven transmembrane receptors). Thus, OR activation triggers an olfactory‐specific G protein (G_olf_) to releases its α subunit into the cilia membrane, which activates adenylyl cyclase. cAMP is a catalytic product, that acts as a second messenger to open a nonselective cation channel known as the cyclic nucleotide‐gated (CNG) channel. The opening of these channels facilitates the influx of cations from outside of the cell, which generates an inward current that shifts the membrane potential toward depolarization. Following the influx of cations, cytoplasmic Ca^2+^ opens Cl_(Ca)_ channels expressed on the cilia, which enables Cl^−^ to flow out of the cell. This further increases the inward current, resulting in signal amplification (Dibattista et al., 2017; Firestein, 1992; Kleene, 1993; Kurahashi & Yau, 1993; Nakamura & Gold, 1987; Reed, 1992; Restrepo et al., 1996). It was suggested that NKCC is required for Cl channels to act excitably and that the co‐transporter is located on the basolateral side of the cell body (Jaén et al., 2011; Reisert et al., 2005). The amplitude of the current depends upon the concentration of the odorant (Kurahashi, 1989).

When an analog voltage change exceeds a threshold, action potentials are generated within the cell body and are projected through an axon extending from the cell body to the olfactory bulb (OB) (Ressler et al., 1993; Vassar et al., 1993), which is part of the brain. Axons from ORCs expressing the same olfactory receptor project to the same site in the OB (Mori & Sakano, 2024), where signals are transmitted through synapses to mitral and tufted cells (Mombaerts, 2004). They subsequently project to various parts of the brain and finally to the olfactory cortex, where smell is perceived. Unlike other senses, olfaction can also access emotions and memories before one recognizes the precise odorant because it innervates into the amygdala, the near hippocampus, and the thalamus before perceived in the olfactory cortex of the brain.

Until recently, all signal modifications for the sense of smell where believed to occur in the brain. It has become clear that at the level of the CNG and Cl_(Ca)_ channels on cilia during the first stage, there is a mechanism in place that influences the perception of smell. In other words, ORCs, as sensory cells, are not merely excitatory cells as information modification occurs much earlier than in the brain. Furthermore, it is influenced by molecules inside and outside of the cilia during the signal generation mechanism. Cilia, which are nanostructures of approximately 100 nm in diameter, have a significant impact on the way we perceive everyday smells. This review summarizes the function of olfactory cilia. In particular, the molecular mechanisms, including the latest findings of three features that are closely related to our everyday sense of smell are discussed, including (1) signal amplification, (2) olfactory adaptation, and (3) olfactory masking.

## 3 SIGNAL AMPLIFICATION BY ION CHANNELS IN CILIA

**FIGURE 2:**
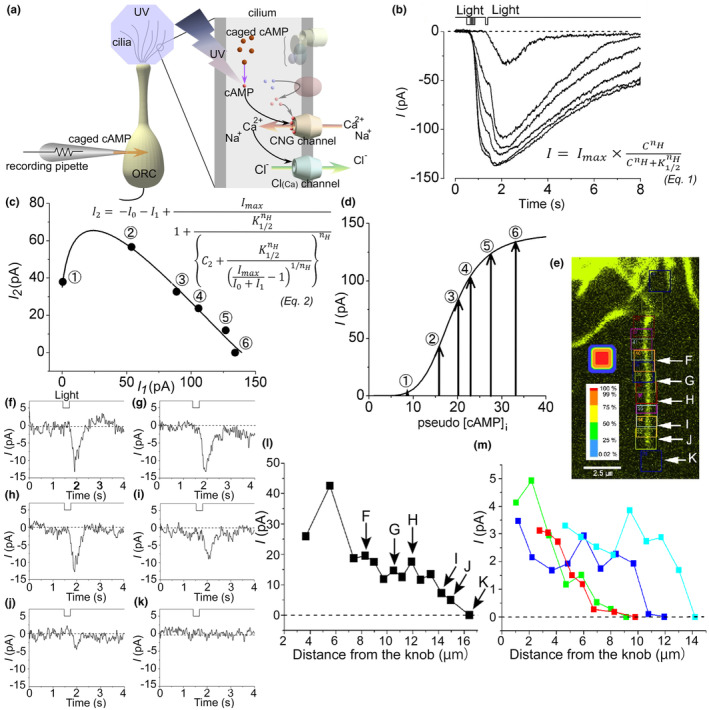
Signal amplification of the current responses. (a) Scheme of signal transduction cascade. cAMP is produced from caged cAMP by UV light photolysis. (b) Current amplification by double‐pulse stimulation in a single ORC. (c) Relationship between the 1st and 2nd response of the light‐induced current. Plots were fitted by Eq. 2 in C, which is based on the Hill equation (Eq. 1). (d) The dose‐response curve was drawn using the same protocol as in C. Arrows indicate the estimated [cAMP]i increased by the 1st light stimuli. (Takeuchi & Kurahashi, 2003, Fig. 6d–f). (e) Local UV stimulation area (colored squares) along the single cilium. (f) Current response from F in e. (g) Current response from G in e. (h) Current response from H in e. (i) Current response from I in e. (j) Current response from J in e. (k) Current response from K in e. (l) Current response from the distance from the knob. (m) Superimposed from four different cilium data (Takeuchi & Kurahashi, 2008, Fig. 5a‐i).

The chemical information of odorants is converted into electrical signals in the cilia (Kleene, 2008; Schild & Restrepo, 1998). During this process, CNG and Cl_(Ca)_ channels are continuously activated. This signal amplification is one mechanism by which even weak odors are detected. First, odorant stimulation triggers the opening of CNG channels and generates a transient inward receptor current, which is followed by depolarization. These two channels each exhibit slight nonlinear amplifications (Lowe & Gold, 1993). When activated sequentially, however, they exhibit strong cooperativity (Takeuchi & Kurahashi, 2002). The odor intensity–response relationship is approximated by the Hills equation, which is a measure of cooperativity. The Hill coefficient is 1–2 for CNG channels alone (Kurahashi & Kaneko, 1991, 1993) and 1–2 for Cl_(Ca)_ channels alone (Takeuchi & Kurahashi, 2005); however, during actual odor response, both channels work continuously to achieve high cooperativity with a Hill coefficient of 3–5 (Figure 2a–d) (Takeuchi et al., 2003; Takeuchi & Kurahashi, 2002).

The ion selectivity of conductance activated by odorants was examined in isolated ORCs. The ion permeability was P_Li_: P_Na_: P_K_: P_Rb_: P_Cs_ = 1.25: 1: 0.98: 0.84: 0.8 (Kurahashi, 1989) and was permeable to all alkali metal ions. Thus, CNG channels are nonselective cation channels. The CNG channel has a four‐subunit structure, CNGA2: CNGA4: CNGB1b = 2: 1: 1. Each subunit has only one cAMP‐binding site, and four molecules of cAMP are required for one CNG channel opening (Zheng & Zagotta, 2004).

The density and distribution of these channels on cilia are important to know when considering the nonlinear signal amplification. With respect to the transduction channel density on cilia, The CNG channels are high densely localized in the cilia (920 channels/μm^2^ in newt, 2400 channels/μm^2^ in toad, Kurahashi & Kaneko, 1991). In the cell body, the CNG channels are expressed at 2 channels/μm^2^ in newt and 6 channels/μm^2^ in toad, respectively (Kurahashi & Kaneko, 1991). Larsson et al. (1997) measured the density of CNG channels in exfoliated cilia specimens from frogs. The current fluctuations associated with CNG channel activation were analyzed in nonspatial clamped cilia, in which the channel density was estimated to be 70 channels/μm^2^. Reisert et al. (2003) reported CNG and Cl_(Ca)_ channel densities of 8 and 62 channels/μm^2^, respectively, in the knob/cilia of rat ORC. They theoretically estimated that CNG and Cl_(Ca)_ channels are located close together on the plasma membrane. Using detached inside‐out cilia preparations and a cAMP diffusion model, Flannery et al. (2006) found that CNG channels are expressed in the first 20% of the cilium in the proximal segments, whereas most of the channels are expressed in the distal segment. Takeuchi and Kurahashi (2008) used a confined laser beam to photolyze caged cAMP or Ca^2+^ in the cytoplasm within the submicron area along the single cilium by photolysis and examined the localization of the photolyzed‐induced currents. The results indicated that the distribution of CNG and Cl_(Ca)_ channels on a single cilium was uniform and widely distributed (Figure 2e–m) at high density.

The presence of Cl channels in the ciliary membrane, which are opened by Ca^2+^, has been demonstrated by the suction inside‐out method (Kleene & Gesteland, 1991) and by caged compounds (Boccaccio & Menini, 2007; Takeuchi & Kurahashi, 2008) for whole‐cell methods. Because the intracellular Cl concentration in ORCs is almost the same as the extracellular Cl concentration, and the equilibrium potential is around 0 mV (Zhainazarov & Ache, 1995), ORCs are considered exceptionally excitatory as nerve cells.

## 4 OLFACTORY ADAPTATION BY CNG CHANNELS IN CILIA

**FIGURE 3:**
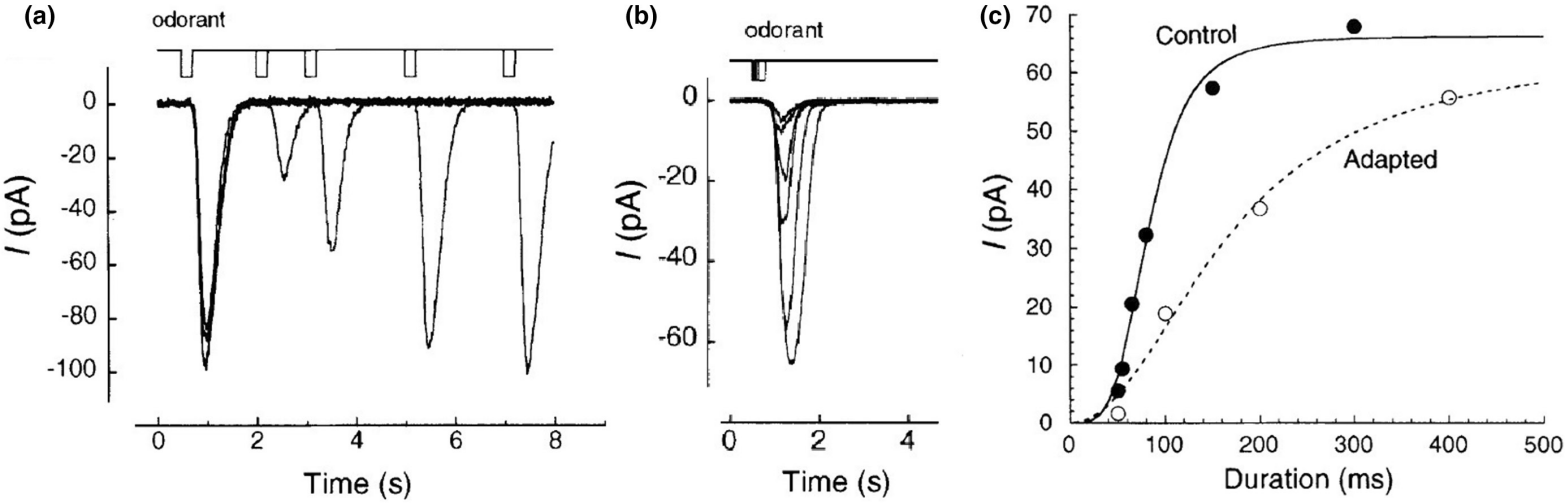
Olfactory adaptation. (a) Double‐pulse odorant stimulation and current response. Time course of adaptation and recovery. (b) Superimposition of the second response. Inter‐stimulus interval times are different, whereas stimulus intensities are the same. (c) Dose–response relationship showing a dynamic shift (Kurahashi & Menini, 1997, figure 1a, c, d).

During the smelling process, inhalation enters the nasal cavity in short pulses. Sudden exposure to a certain odor may be very strong initially, but soon the individual becomes accustomed to the odor and does not smell it as much. This phenomenon occurs when we no longer perceive the smell, although the odorant remains in our nose. It was once thought that this was a modification occurring in the brain, but it occurs at the cilia. This is called “olfactory adaptation”. Calcium ions in the cilia are deeply involved in this phenomenon. The perception of odors becomes less intense when the same odor is repeatedly sniffed, or a weakened perception occurs when the same odor is sniffed immediately after a single sniff (a few seconds). Although we are familiar with these phenomena, the definition of adaptation is often misunderstood. Some individuals consider the gradual decrease in response to a sustained stimulus to represent adaptation; however, this is incorrect if Ca^2+^‐dependent adaptation at the ORC level is considered. When double‐pulse odorant stimulation is considered, the second responses are smaller compared with the first (Figure 3a,b) (Kurahashi & Menini, 1997). Adaptation at the ORC level refers to the ability to broaden the concentration range (dynamic range) of the response. The dose–response relation shifts to the right based on the stimulus intensity, thereby broadening the range of stimuli that can be perceived (Figure 3c). This mechanism enables the ORCs to perceive odor at very strong concentrations and to notify the brain of differences in concentration. Olfactory desensitization indicates a decrease in the saturation of the response. For olfactory adaptation, this does not mean that we no longer perceive the odor, instead, we are preparing for the next strong odor to appear.

This phenomenon is caused by Ca^2+^ influx into the cilia through CNG channels before signals are sent to the brain. The Ca^2+^‐dependent adaptation target is most likely the ciliary CNG channel (Chen & Yau, 1994; Kurahashi & Menini, 1997). Current suppression occurs following Ca^2+^ influx through the CNG channel, Ca^2+^ then binds to calmodulin and the Ca‐CAM complex blocks the CNG channels, where it remains bound for some time. In isolated ORC experiments, adaptation occurs in a few seconds, and recovery from adaptation takes approximately 10 s (Figure 3a). We perceive that our sense of smell recovers from adaptation in approximately 10 s; however, other olfactory adaptation systems must be considered, not only in ORCs, but also in the higher center of olfaction reception, such as the brain.

## 5 FUNCTIONAL ANALYSIS OF CALCIUM SIGNALING IN THE NANOSCALE OLFACTORY CILIA

**FIGURE 4:**
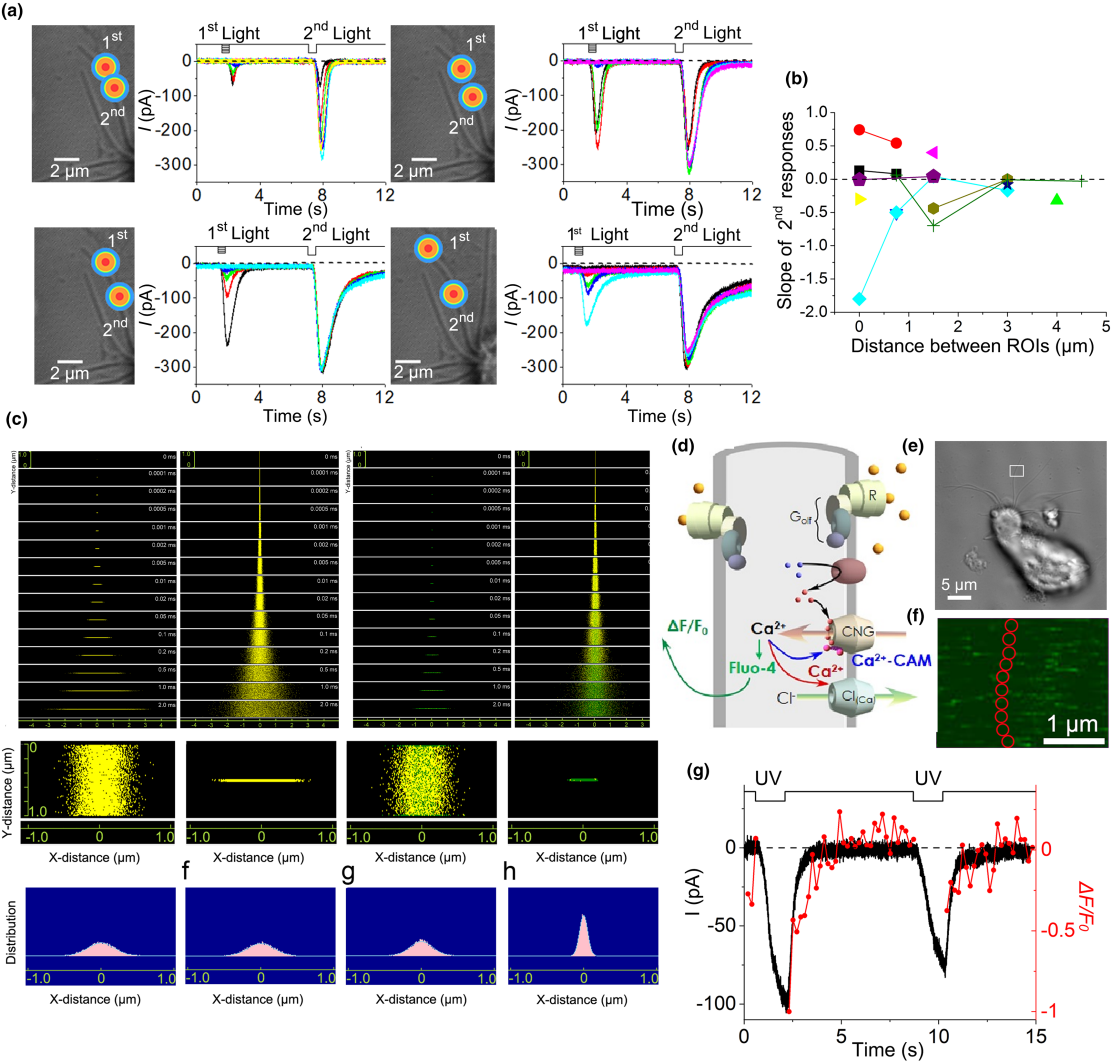
Diffusion limitation in the cilium. (a) Double‐light pulse stimulation. Left: Double‐pulse stimulation points. Right: Current response (Takeuchi & Kurahashi, 2018, figure 5a‐h). (b) Distance between two stimulus points and the slope of the dose–response relationship. A decrease in slope angle indicates adaptation from calcium ion transfer, whereas an increase in slope angle indicates addition resulting from cAMP transfer. Eventually, the slope angle reaches zero within a few μm. (Takeuchi & Kurahashi, 2018, figure 5j) (c) Simulation of the molecules in the tiny structure (Takeuchi & Kurahashi, 2018, figure 9–11a‐h). (d) Scheme of simultaneously measurements channel current and Fluo4 fluorescent. (e) Photograph of the single ORC. White box shows scan area. (f) UV stimulation area in white box in e. (g) Superimposition of the current response and fluorescence intensity for the double‐pulse stimulus (Takeuchi & Kurahashi, 2023, figure 6h).

Recently, it was reported that both signal amplification and adaptation occur in the same nanoscale space of the cilia (Takeuchi & Kurahashi, 2023). Molecular movement is limited in the narrow space in a single cilium (Figure 4a), and diffusion limitation is within a few microns distance (Figure 4b). This phenomenon was studied not only in cell experiments, but also using techniques, such as digital simulation and calcium imaging (Figure 4c,d). In a series of experiments, cytoplasmic Ca^2+^ dynamics in the single cilium were used to explain signal amplification and adaptation (Figure 4e‐g). Olfactory cilia have a characteristic morphology, which consists of a microstructure with a diameter of 100 nm and a length of 10 μm or greater depending on the animal species. This has the advantage of (1) acquiring a high surface area/volume ratio and (2) increasing the probability of binding to odor molecules. This may facilitate signal transduction to increase efficiency. Because of the expression of ORs, enzyme groups, channel groups, and other proteins on cilia, the ciliary membrane is crowded. Therefore, if the volume is small and the variation in the number of molecules is limited, the S/V ratio will be high because of the narrow diameter of the cilia. Even a small number of molecules in the cilia can affect factors in the cascade and result in large concentration changes.

Olfactory signaling is mediated by chemical reactions that proceed for approximately 1 s via cAMP. Ca^2+^ flows into the cilia from outside of the cell through an opening in the CNG channels and accumulates. This supports two major functions of olfaction: signal amplification (Section 2) and adaptation (Section 3). Signal amplification occurs through the opening of a Cl_(Ca)_ channel, whereas adaptation is regulated by Ca^2+^ feedback to CNG channels. Thus, Ca^2+^ influx and the concomitant increase in cytosolic Ca^2+^ levels result in opposite effects by increasing channel currents, whereas adaptation decreases channel currents. These two functions are often interpreted as counteracting one another. Using patch clamp and fluorescent imaging of Ca^2+^ at a local part of a single cilium, cell data suggested that the current response and Ca^2+^ concentration change were handled by the Ca^2+^ time course. (Takeuchi & Kurahashi, 2018). In fact, within living cilia, Ca^2+^ is used in a small space. Although the two systems appear to contradict one another; this is accomplished by the time difference between their activities. The rapid signal amplification response followed by the slow response of adaptation indicates that Ca^2+^ within cilia has two roles (Takeuchi & Kurahashi, 2023).

## 6 OLFACTORY MASKING BY SUPPRESSION OF CNG CHANNELS IN CILIA

**FIGURE 5:**
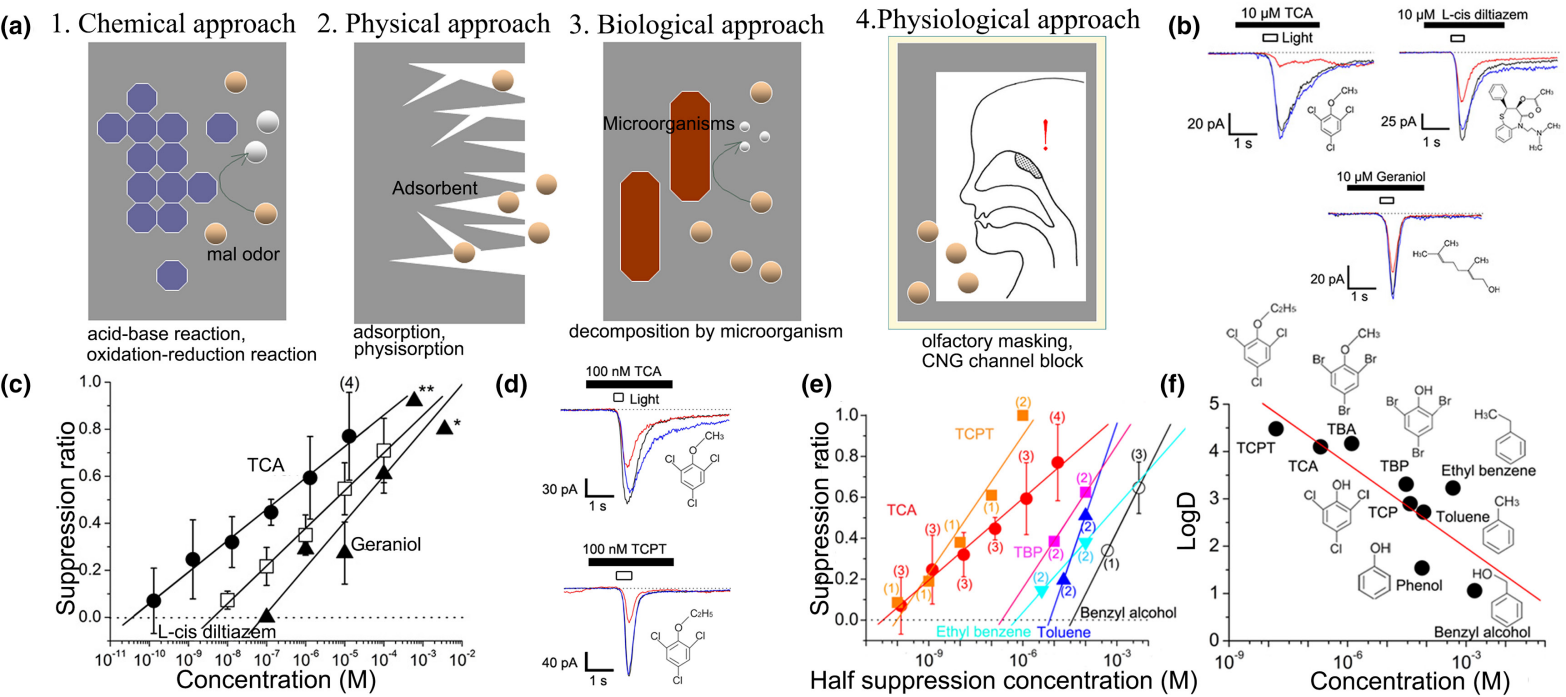
Olfactory masking. (a) Three deodorizing approaches and a new concept: Olfactory masking. (b) Current suppression by TCA and suppressors. (Takeuchi et al., 2013, figure 2a‐c). (c) Dose‐suppression relation in b. (Takeuchi et al., 2013, figure 2d). (d) Current suppression by TCA and TCPT. (Takeuchi et al., 2013, figure 6a, b). (e) Dose‐suppression relationship among suppressors in d. (Takeuchi et al., 2013, figure 6c). (f) Relationship between LogD (pH7.4) and half‐suppression concentration (Takeuchi et al., 2013, figure 6d).

Olfactory masking is a mechanism to attenuate responses to extracellular odorants and has only recently been elucidated. It is often misconstrued as deodorization or deodorant. There are three “deodorizing approaches”: (1) chemical, (2) physical, and (3) biological. Chemical approaches use a catalyst to break down an odorant into different substances to eliminate the bad odor. Physical approach uses a method to reduce the absolute number of molecules suspended in the air by adsorbing the odorants with a substance containing a large surface area (e.g., charcoal or gel). Biological approach employs a method to decompose odorants using microorganisms. All three approaches have the same target, which are malodorous substances; however, the concept of olfactory masking as new approach is completely different, and the target is the CNG channels in the nose (Figure 5a).

The difference between malodor and aromatic odor sensitivity markedly varies from person to person and is controlled by the brain. There is no difference between malodors and fine aromas at the ORC level because the ORs recognize the various odorants. The cascade that follows is just one pathway common to all odorants. One study is reported that the odorant‐induced current is suppressed by the odorant found at the single ORC level (Kurahashi et al., 1994). Odorants can excite ORCs and suppress the response simultaneously. Which part of the ORC suppresses excitation? Chen et al. (2006) found that the suppression target is the CNG channels, which occurs through the formation of homo‐ or hetero‐oligomeric CNG channels. The results of electrophysiological experiments and sensory tests indicated that CNG channel inhibition by each odor molecule was consistent with human olfactory output (Takeuchi et al., 2009). This indicates that the true nature of olfactory masking is the suppression of CNG channels by odor molecules. Thus, humans are less sensitive to smells during olfactory masking, even when they are present.

This olfactory masking is the mechanism by which extracellular odorants, present in the mucus layer, reduce the activity of CNG channels from outside of the cell. The rate of current reduction depends on the odorant and is concentration‐dependent. Currently, the most potent known masking agent is TCA (2.4.6‐trichloroanisole) with an EC_50_ of 0.19 μM (concentration in the puffer pipette). L‐cis diltiazem is a CNG channel‐specific inhibitor with an EC_50_ of 5.8 μM, whereas geraniol is also a natural masking agent (29 μM) (Figure 5b,c). It was also shown that 10 aM (10^‐18^ M) of TCA inhibited the inward current when the concentration was precisely controlled. A 10 aM solution contains approximately 800 molecules of TCA in a 1 mL solution, whereas the number of CNG channels inhibited is approximately 10,000 (Takeuchi et al., 2013). This suggests the possibility of a one‐to‐many, rather than a one‐to‐one blockade relationship. The molecular mechanism of inward current inhibition at very low concentrations in unclear. The high LogD values and the chemical structure suggest that masking agents may dissolve into the ciliary membrane and affect the channels (Figure 5d‐f). The mystery of cork taint caused by a very small amount of TCA in wine may be related to this suppression (Takeuchi et al., 2013). Certain pesticides (e.g., chlorpyrifos) (Takeuchi & Kurahashi, 2019) inhibit CNG channels, resulting in a significant loss of flavor in bananas.

These findings indicate that olfactory masking reduces our sense of smell, despite the presence of odorants in our surroundings. In turn, this loss of olfactory perception results in the loss of flavor. If the original flavor is pleasant, a reduction of smell causes an unpleasant effect. At the same time, this suppression may be used for the reduction of malodors. Pleasant aromas increase appetite and promote mental health, including improving quality of life. Finding ways to effectively use and control olfactory masking may significantly change our lives.

## 7 CONCLUSION

Olfaction is no longer a fuzzy sense. Electrophysiology has been applied to ORCs, quantitative analysis of real‐time responses has become possible, and the olfactory transduction system, once thought to be complex, has gradually become clearer. Moreover, the molecular structures of the ion channels and membranes may be studied through single‐molecule measurements and super‐resolution imaging using a wide variety of cells. Cilia, which are the site of olfactory signal transduction, contain very fine structures with a diameter of 100–200 nm and a length of approximately 10 μm. Thus, it is a challenge for them as a living experimental sample. Nevertheless, many processes can only be understood with native samples. The development of a variety of experimental techniques and electrophysiology will lead to further discoveries in the field of olfaction.

## AUTHOR CONTRIBUTIONS

HT wrote the manuscript.

## FUNDING INFORMATION

Japan Society for the Promotion of Science (JSPS), KAKENHI: Hiroko Takeuchi 19 K06764; Nakatani Foundation for Advancement of Measuring Technologies in Biomedical Engineering (Nakatani Foundation), Grant for Development Research: Hiroko Takeuchi 2023K104.

## CONFLICT OF INTEREST STATEMENT

Not applicable.

## ETHICS STATEMENT

Not applicable.

## PERMISSION TO REPRODUCE MATERIAL FROM OTHER SOURCES

1. Originally published in the Journal of General Physiology. https://doi.org/10.1085/jgp.200308911 (Figure 2b–d)
2. Originally published in the Journal of General Physiology. https://doi.org/10.1085/jgp.201812126 (Figure 4a–c)
3. Originally published in the Journal of General Physiology. https://doi.org/10.1085/jgp.202213165 (Figure 4d–g)
4. Copyright (2013) National Academy of Sciences (Figure 5b,c)
5. Copyright (2008) Society for Neuroscience (Figure 2e–m)

## Data Availability

Encourages: Data Sharing.
